# A Study Investigating How the Characteristics of High Reliability Organisations Can Be Measured in the Construction Industry in Australia

**DOI:** 10.3390/ijerph17218273

**Published:** 2020-11-09

**Authors:** Andrew Enya, Shane Dempsey, Manikam Pillay

**Affiliations:** 1 School of Health Sciences, University of Newcastle, Callaghan, NSW 2308, Australia; Shane.dempsey@newcastle.edu.au (S.D.); Manikam.pillay@newcastle.edu.au (M.P.); 2 Centre for Resources Health and Safety, University of Newcastle, Callaghan, NSW 2308, Australia

**Keywords:** high reliability organisation, safety, collective mindfulness, construction industry

## Abstract

Construction activities involve a lot of risk as workers are exposed to a wide range of job hazards, such as working at height, moving vehicles, toxic substances, and confined spaces. The hazards related to a construction project are mostly unpredictable because construction projects move quickly due to project deadlines, and changing work environments. As a result of this, the industry accounts for one of the highest numbers of work-related claims, and the fourth highest incidence rate of serious claims in Australia. This research investigates how key safety management factors can measure the characteristics of high reliability organisations (HROs) in the construction industry in New South Wales Australia. To address the problem, a model is presented that can predict characteristics of HRO in construction (CHC). Using structural equation modeling (SEM), and confirmatory factor analysis (CFA), the model and measurement instruments are tested and validated from data collected from construction workers. The results identified the factors that effectively measure CHC, and the findings can also be used as a safety management strategy and will contribute to the body of knowledge in research.

## 1. Introduction

In the attempt to improve work health and safety, Safe Work Australia developed a strategy that seeks to influence key national activities in all industrial sectors, with more focus on sectors with increased rates of injuries and fatalities. The strategy is also targeted towards policy makers, regulators, and those in the position to improve work health and safety in the workplace across Australia [1].

To achieve the Strategy, three national targets must be attained by 2022, which are; (i) a reduction in the number of worker fatalities due to injury of at least 20%, (ii) a reduction in the incidence rate of claims resulting in one or more weeks off work of at least 30%, and (iii) a reduction in the incidence rate of claims for musculoskeletal disorders resulting in one or more weeks off work of at least 30% [1]. While the strategy applies to all the industrial sector, it focuses on seven key industries which includes the construction industry. The construction industry is included because it accounts for one of the highest numbers of work-related claims and reported the fourth highest incidence rate of serious claims in Australia from 2014–2018 [2].

There is a conception that risks are embedded in construction activities, and safety management competes with production [3,4], hence they are incompatible goals. This conception has resulted in the consistent emphasis by researchers that the construction industry is a high-risk industry which needs result-oriented approaches to manage construction safety [5]. Construction activities are embedded with lots of risk as workers are exposed to a wide range of job hazards, such as working at height, moving vehicles, toxic substances, and confined spaces. The hazards related to a construction project are mostly unpredictable because construction projects move quickly due to project deadlines, and changing work environments, so it is not always easy to maintain a safe and stable working environment [6]. These changes in the work schedule and pressure to meet deadlines makes the industry complex, and the complexity is one thing they have in common with high reliability organisations (HROs) [5].

Reliability seeking organisations such as construction, oil and gas, health care, and manufacturing are confronted with the problem of which attributes of HRO can be transferred and measured to improve organisational safety management. The present research has either focused on literature reviews of HRO applicability in various organisations [7,8,9], while others have conducted empirical studies to test and validate measurement instruments, and investigate conceptual frameworks [10,11,12,13,14,15,16,17]. A few measurement instruments have become available in the last few years, with most of them being developed and validated in the health care sector, based on Weick’s and Sutcliffe’s [18] principles of collective mindfulness. Other studies conducted have proposed frameworks that can be used for pain management in healthcare [19], identified the presence and application of HRO in the oil and gas industry, construction, crowd management, business school, and disaster prevention [12,14,16,17]. However, only three studies have empirically investigated HRO in the construction industry [4,17]. The first study by Hoyland et al. [17] identified the presence of HRO safety principles in the health care sector and construction industry in Norway. The study used semi-structured interviews for data collection on a small sample size of eight participants. The study had some limitations such as, the small sample size and the participants for the construction industry study were apprentices and young workers with limited work experience [17]. The second study by Mitropoulos and Cupido [4] investigated the practices of high-reliability crews (HRC) contracted to carry out framing work in the residential construction industry. The study revealed that the HRCs, in comparison with an average-performing crew, had a strong and clear guiding principle and a set of strategies that focused on preventing errors and rework [4,20]. The third study [21] was conducted in Australia across six organisations, including the construction industry and measured mindfulness of work health and safety in the workplace. The findings from the study suggest that employers in construction, manufacturing, accommodation, and food services were more mindful of work health and safety, and both employers and employees had a high level of mindfulness towards workplace safety based on their respective job role. While the study had a large sample size, most of the respondents were from small businesses or were sole traders (self-employed), and only two principles of collective mindfulness was measured (preoccupation with failure and sensitivity to operations) [21]. Despite the advancement in research, there is the absence of empirical research that measures attributes of HRO in the construction industry and how such attributes can improve construction safety management. There is also the problem of a knowledge gap as there is no guiding framework on what aspect of HRO that can actually be beneficial in improving safety management.

HROs are known to exhibit strong commitment to safety, they have decentralised decision making within a flexible management hierarchy. They do not have the luxury of trial-and-error learning because mistakes can result in severe consequences, therefore they depend on procedures that will avoid failures [19] by combining persistent cognitive processes to manage unexpected events effectively [22]. The presence of powerful cultures of learning, openness, and accountability in HRO [9] are characteristics that makes it a viable approach to improve construction safety. Aspects of HROs have been identified by Weick and Sutcliffe, but methods to implement them are rarely available or absent [22,23,24,25,26,27,28]. The low number of HRO research is an indication that there is the need for further empirical investigation of HRO in the construction industry. In addition, research has suggested that HRO enhancing practices could be transferred to the construction industry and implemented as a safety management strategy [4,5,20].

To address the research problem, an empirical study was conducted to investigate the characteristics of HRO present in the construction industry in Australia, through the use of an integrative model. The model uses key safety management factors to measure the characteristics of HRO, and provides better understanding of how the characteristics can improve construction safety management. The paper is structured as follow. A review of HRO and its characteristics are presented, which provides the background for the development of the integrative model. Then, the methods used to empirically test the measurement instrument and model are described, followed by the results. Then, the discussion of the results, implication for construction safety management, research limitations, and conclusion are presented.

### 1.1. Literature Review

High reliability organisations (HROs) are known to operate in extremely hazardous environments where the potential for failure is present, and to avoid failures, they use systematic safety processes to manage complex technology. Their operations are tightly coupled (i.e., systems are closely connected and depend on each other for effective operation) as they cannot afford the luxury of learning by experimentation [22,29]. They are also able to find the balance between safety and production in their operations. In the construction industry, accidents and near misses are managed using safety management procedures such as; permit-to-work system, job safety analysis (JSA), safe work method statements (SWMS) [30], incident and near miss reporting procedures, and risk assessment. These systems of safety management in construction have some similarities with HRO characteristics, but much has not been done in terms of research to empirically identify the characteristics that can improve construction safety management.

Five key characteristics found in HROs are: preoccupation with failure, reluctance to simplify, sensitivity to operation, commitment to resilience, and deference to expertise [27].

#### 1.1.1. Preoccupation with Failure

Preoccupation with failure involves a constant feeling of unease, even when it appears that all is well, operations are focused on the likelihood that unforeseen events can disrupt operational safety, so to prevent it, HROs engage in proactive analysis of weak signals by continuously seeking errors and lapses that have the potential to lead to the failure of the system [31]. They are able to avert failures through effective reporting of near misses and errors and implementing the lesson learnt [32].

#### 1.1.2. Reluctance to Simplify

HROs have a mental mindset where they try to maintain thinking at a conceptual and abstract level in order to manage the unexpected by deliberately questioning assumption and received wisdom, to create a clear and understandable picture of current situations, by simplifying less and seeing more [25,31].

#### 1.1.3. Sensitivity to Operations

This involves maintaining the ‘bigger picture’ of operations by actively seeking the views of front line staff to get accurate representation of the status of operations. Reliable information is communicated about potential human and organisational failures, and changes are implemented to prevent the accumulation of errors and escalation of events [31].

#### 1.1.4. Commitment to Resilience

This is an organisation’s ability to effectively anticipate errors and also be able to manage the outcome and bounce back from such errors [31]. HROs are able to successfully recover from errors because of their capability to learn from past incidents [31].

#### 1.1.5. Deference to Expertise

This is the process of allowing the most experienced people to make safety critical decisions during emergencies despite their rank in the organisational hierarchy. Afterwards, decision making reverts back to organisational hierarchy [24].

### 1.2. Conceptual Model (Key Safety Management Factors)

**Figure 1:**
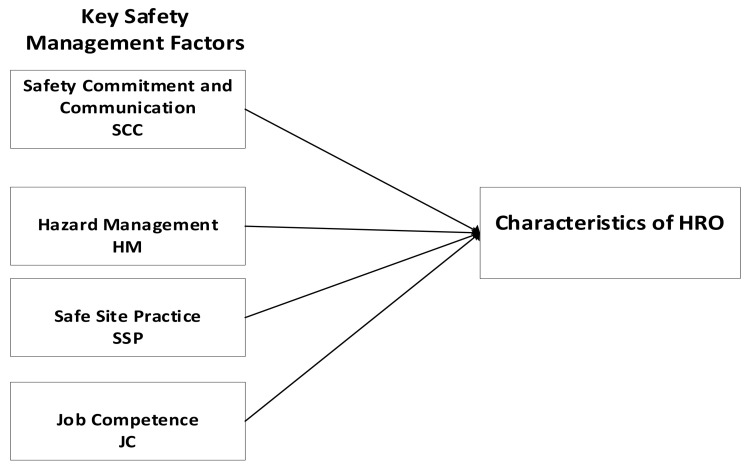
The proposed model to identify and measure characteristics of High Reliability Organisations (HROs) present in construction.

The model for this study (see Figure 1) was developed from an extensive literature review of seminal work and recent empirical studies on HRO [22,26,27,33]. Key safety management factors that are associated with the HRO characteristics were identified, and used to develop the model. The factors are: safety commitment and communication, hazard management, safe site practice, and job competence [22,25,26,28]. The factors are then linked to the characteristics of HRO they measure. Further details of the model testing and validation is provided in the Methods section. The next section describes the factors.

#### 1.2.1. Safety Commitment and Communication

Safety is top priority in the operations of HROs, which gives them the ability to find the balance between safety and production. Their operational reliability is achieved by developing highly standardised work routines and employing competent and well-trained staff [34]. Communication is the sharing of information through a two-way process where a person listens to what others have to say and understands what it means, and the person is also listened to in return. Vital information is easier to discuss when people communicate effectively in the workplace, as it gives an opportunity to understand the perspective of others, and better decisions are made [35]. Safety commitment and communication accesses construction organisations commitment towards providing a safe working environment for their employees and contractors and the procedures they follow to communicate safety information on construction sites. This factor measures preoccupation with failure, reluctance to simplify, and sensitivity to operations.

**Hypothesis 1:**  *Perception of construction workers safety commitment and communication predicts characteristics of HRO in construction.*

#### 1.2.2. Hazard Management

This is an organisation’s ability to identify and be aware of hazards in the workplace both at the organisational and individual level, accidents and near misses are reported and the outcome from the investigation used as a learning point to improve hazard management and overall safety management [32]. This accesses the strategies organisations implement to improve their hazard management, and measures preoccupation with failure, sensitivity to operation, commitment to resilience, and deference to expertise.

**Hypothesis 2:**  *Perception of construction workers hazard management predicts characteristics of HRO in construction.*

#### 1.2.3. Safe Site Practice

Safe site practices are the procedures implemented by organisations to ensure that workers follow all safety procedures when performing their duties. This is implemented through the use of the safe work method statement and effective supervision [30]. This measures sensitivity to operation, commitment to resilience, and deference to expertise [36].

**Hypothesis 3:**  *Perception of construction workers safe site practice predicts characteristics of HRO in construction.*

#### 1.2.4. Job Competence

This involves the process organisations follow to ensure they employ competent and experienced workers. This is important so that they can work safely and are able to make good decisions when they face challenges. HROs have a decentralised decision process making which allows the most experienced and qualified staff to make decisions instead of leaving it or waiting for top management, especially when it relates to workplace and job safety, hierarchy is reverted to normal afterwards. Job competence measures reluctance to simplify, commitment to resilience, and deference to expertise [30].

**Hypothesis 4:**  *Perception of construction workers job competence predicts characteristics of HRO in construction.*

## 2. Materials and Methods

### 2.1. Study Design

This study involved a cross-sectional survey of frontline managers and frontline construction workers in Newcastle, New South Wales, Australia, identified and measured the characteristics of HRO present in the construction industry. The University of Newcastle Human Research Ethics Committee granted ethical approval for the study (H-2018-0330).

The survey instrument was developed specifically for the study based on relevant HRO literature [12,14,16,22,27,34,37], and the survey included 32 questions and contained self-assessment items measured on a 5-point Likert type scale from “strongly disagree” to “strongly agree”. The survey consisted of two parts. Part one included demographic questions and was aimed at identifying (job role, years of experience in organisation, and years of experience in industry). The second part consisted of four sections that make up key safety management factors in construction. Safety commitment and communication (SCC) (11 items), hazard management (HM) (7 items), safe site practice (SSP) (7 items), and job competence (JB) (3 items). The questions for part two, Section 1, Section 2, Section 3 and Section 4 were developed using Weick and Sutcliffe’s (2001) 47-item questionnaire [25], and Youngberg’s self-assessment tool as a reference in developing questions that were specifically about characteristics of HRO [34], and the questions were revised to ensure it made sense in the construction industry context.

Section 1, Section 2, Section 3 and Section 4 consisted of questions about the aspects of construction safety management targeted at measuring HRO characteristics. Section 1 (safety commitment and communication) measured preoccupation with failure, reluctance to simplify, and sensitivity to operation. Section 2 (hazard management) measured preoccupation with failure, sensitivity to operation, commitment to resilience, and deference to expertise. Section 3 (safe site practice) measured sensitivity to operation, commitment to resilience, and deference to expertise. Section 4 (job competence) measured reluctance to simplify, commitment to resilience, and deference to expertise.

To establish face and content validity, the questions were reviewed twice and modified by an expert panel that included a safety advisor working in the construction industry and three occupational health and safety academics with HRO research experience. Subsequently, some questions were reworded based on feedback from the panel which resulted in the development of a 32-item cross-sectional survey that contained 4 demographic questions and 28 scale items.

The reliability score for the instrument was α = 0.867, according to Hair et al. [38], internal consistency for data is high when Cronbach’s alpha is more than 0.7, therefore the reliability of the scale was above the minimum criteria.

### 2.2. Participants and Organisation Recruitment

Purposive sampling was used to ensure that participants were able to provide specific information to address the research question, have an understanding of the topic being investigated, and also include two specific hierarchical levels within the organisation [39]. Middle managers and frontline workers were selected to capture the perception of construction workers with the minimum and extensive working experience.

Participant recruitment for the study was two phased; the first phase was for the selection of organisations, while the second phase was for participants. Organisations were contacted if they were involved in large scale commercial projects and mid-sized infrastructure projects and have been operating for a minimum of five years continually. This was done to ensure that the organisations had a structured and functional safety management system capable of managing high-risk activities, and incident investigation. For participants to be eligible for the study, they were required to have a minimum of two years working experience in the industry, be more than 18 years old, and involved in or supervise high risk construction activities (working at height, confined space, etc.). Participants involved in high risk construction activities were chosen because those activities are known to cause most accidents on construction sites [30]. Emails containing letters of invitation and an information statement were sent to 50 purposively selected construction organisations in Newcastle and the Hunter region, with follow-up phone calls and site visits. At the end of the first phase, seven construction organisations accepted to take part in the study. The second phase involved site visits to the participating organisations to meet with the gate keepers (site managers and supervisors) and potential participants to create more awareness on the research and what was required of them. Potential participants were invited to participate during subsequent site visits and through research advertisement posters placed in their workplaces.

### 2.3. Data Collection

Data were collected using a survey questionnaire distributed to 384 construction workers across seven organisations during toolbox meetings and safety training. Participants were provided with an information statement, survey questionnaire, and consent form, and participation was totally voluntary and anonymous. Two boxes were provided at each participating organisation for returning completed questionnaires and consent forms, which were picked up weekly by the first author throughout the duration of the survey (June 2019–December 2019). A total of 250 survey questionnaires were completed. Two-hundred and ten were correctly completed and 40 were incomplete, representing a response rate of 54.6%.

### 2.4. Data Analysis

Data analysis was conducted using Statistical Package for Social Sciences (SPSS) version 25 (IBM Corp., Armonk, NY, USA). Creswell [40] recommends descriptive analysis be conducted for all variables and the results presented to include means, standard deviation, and range of scores [40]. In regards to this, univariate analysis was conducted for descriptive statistics such as frequency, means, percentage, and standard deviation for the participants and organisation demographics, and all variables. The characteristics of the HRO score were calculated by summing the key safety management factors.

Exploratory factor analysis (EFA) was conducted to examine the factorial structure of the measurement instrument. This was done to ensure that items measuring the same construct would significantly load on the same factor instead of loading on other factors. EFA discovers the number of factors influencing a variable by summarising patterns of correlation between observed variables while reducing a large number of observed variables to fewer number of factors [41,42]. The Kaiser–Meyer–Olkin (KMO) measure and Bartlett’s test of sphericity were conducted to assess the suitability of the respondent data for factor analysis [43]. Principal component analysis (PCA) was conducted using the Promax rotation in IBM SPSS 25 (IBM Corp., Armonk, NY, USA)

The structural equation modelling (SEM) procedure was also used for data analysis. SEM is an efficient method for hypotheses testing of relations in observed and latent variables [44], it also performs factor analysis and path analysis simultaneously because of its ability to measure and accommodate errors of manifest variables [45]. There are two methods of SEM available to researchers to choose from, these methods are: covariance based SEM (CB-SEM) [46,47], and the partial least square SEM (PLS-SEM) [47,48]. The statistical aim of CB-SEM is to estimate model parameters that minimises the difference between the observed sample covariance matrix that is calculated before a theoretical model solution is achieved. Additionally, when the goodness of fit (GOF) is established, the model is accepted and if GOF is not established, the model is not accepted [47,49]. While the statistical aim of PLS-SEM is to maximise the variance explained in the dependent variable, with focus on optimising the prediction of the endogenous constructs and not on fit [47,50].

SEM was adopted for this study as it enables the researcher to explore characteristics of HRO in construction, test the measurement model and the integrative model proposed for this study. CB-SEM was adopted to test the measurement model for this study using confirmatory factor analysis (CFA). CFA was conducted in the computer program AMOS v25 (IBM SPSS, Chicago, IL, USA) with maximum likelihood (MLE) estimation and the covariance matrix [51]. PLS-SEM was adopted to test the proposed structural model and hypothesis. Structural model assessment was carried out in the computer program SmartPLS (v. 3.3.2) [52].

## 3. Results

A total of 210 correctly completed survey questionnaires were used for data analysis, representing a response rate of 54.6%. The results from the analysis are presented in two parts, part one presents descriptive statistics of demographics and summary of the survey results, part two presents the results of the exploratory factor analysis, confirmatory factor analysis, structural model assessment, and hypothesis testing.

### 3.1. Part 1

#### 3.1.1. Participants Characteristics

The demographics and characteristics of the participants are presented in this section. Table 1 displays the participant’s job role and their staff type. Frontline workers were (57.6%) with various job roles illustrated in the job role section of Table 1, while (42.40%) were frontline managers whose job roles are also displayed in the job role section.

Table 2 shows the years of working experience of participants in their organisation and construction industry. Organisation experience refers to experience participants had in the organisation they were employed in when they took part in the survey. Participants were required to have a minimum of two years construction experience to participate in the survey, and 84 out of 210 had less than five years’ experience, and the remaining 126 had more than five years’ experience, therefore all 210 participants qualified to take part in the survey.

#### 3.1.2. Organisation Characteristics

Seven construction organisations participated in the study, three were large organisations employing more than 200 staff, and four medium organisations employing 50–200 staff. Table 3 shows the type of organisation, type of project embarked upon, and the number of middle management and frontline workers that participated from each organisation.

#### 3.1.3. Summary of Survey Scores of Respondents

The scores of the variables from the participant’s responses were calculated and to identify the factors that had the highest scores. The scores of the four variables were summed up to get the characteristics of HRO in the construction score. Summary of the results are presented in Table 4.

Safety commitment and communication scored the highest, followed by safe site practice. Frontline managers had the highest scores for all the measured factors, although the difference of the scores between both staff group was not so much. This result compares with previous studies and confirms the fact that as workers move up organisational hierarchy, their attitude towards safety increases [7,9].

### 3.2. Part 2

#### 3.2.1. Exploratory Factor Analysis 

Principal component analysis was conducted in SPSS 25 (IBM Corp., Armonk, NY, USA) on the 28-item questionnaire that measured characteristics of HRO on 210 construction workers. PCA was accessed for its appropriateness before commencing the analysis. The Kaiser–Meyer–Olkin (KMO) index ranges from 0 to 1, with 0.05 considered suitable for factor analysis [43]. The KMO measure was 0.77, exceeding the recommended value. The Bartlett’s test of sphericity was statistically significant (chi square value = 2702.404, *p* value = 0.000), which suggested that the data were suitable for factorisation. An initial unforced PCA on the key safety management items revealed nine factors with an eigenvalue greater than one from the 28 questions explaining a total variance of 69.64%. The number of extracted factors was then set to four as the study was interested in four constructs to measure characteristics of HRO. A total variance of 56.04% was explained for the four factor solution. A cut off factor of 0.40 loading was applied, variables were retained if they had a loading of ≥0.40, and a difference between loadings that exceeded 0.02 [53]. Some items were dropped (SCC4, SCC6, SCC7, SCC9, HM4, HM5, SSP5, SSP6, and SSP7) because of their low communality values and did not meet the recommend factor loading of 0.40 [54].

A final PCA with Promax rotation was conducted to assist in interpretability and achieve an optimal factor structure [41]. Interpretation of the questionnaire items was consistent with the characteristics of HRO it was designed to measure, it had strong loadings for hazard management items in factor 1, safety commitment and communication items on factor 2, safe site practice on factor 3, and job competence on factor 4. The KMO for the identified factor structure was 0.717 with a statistically significant Bartlett’s test of sphericity (chi square value = 1420.678, *p* value = 0.00). Factor loadings of the rotated solution are presented in Table 5. The factor structure was used for the CFA in the next step of the research.

#### 3.2.2. Testing of the Measurement Model

**Figure 2:**
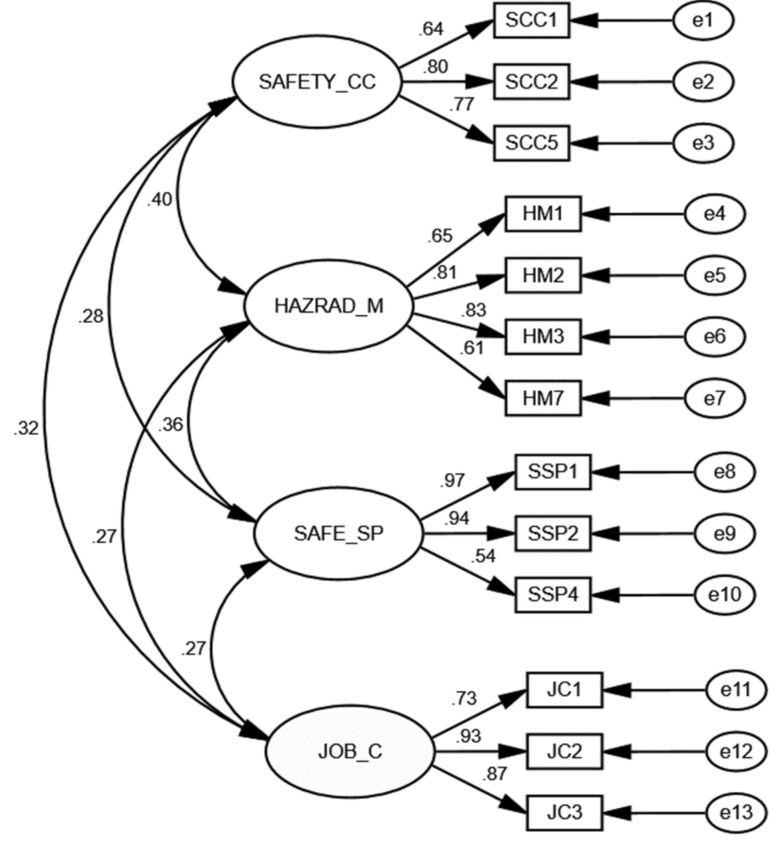
Standardised estimates for the 13-item four-factor structure.

Confirmatory factory analysis (CFA) was conducted in AMOSv25 (IBM SPSS, Chicago, IL, USA) with the maximum likelihood estimation and covariance matrix to test the measurement model. In assessing the fitness of a model, there are three model fit indices that are used: absolute fit (GFI, RMSEA and chi-square), incremental fit (AGFI, CFI, TLI, NFI), and parsimonious fit (Chisq/df). Goodness of fit (GFI), comparative fit index (CFI) and incremental fit index have values that range from 0 to 1, with values greater than 0.90 indicating a good fit [54]. RMSEA should be less than 0.06 and SMRM should be less than 0.08. In examining the strength of scale structure, it is recommended to consider multiple fit indices [55]. This study used CFI, RMSEA, and SMRM as measures to examine model fit. The modification indices was inspected to improve the model fit measures, as a result of the inspection six items from three factors (four from safety commitment and communication, one from hazard management, and one from safe site practice) were dropped from the CFA as they had poor loading factors. The result of the model including four factor and thirteen indicators showed excellent fit (chi-square/df = 2.124, CFI = 0.952, RMSEA = 0.073, SRMR = 0.075). All items factor loadings were statistically significant (*p* < 0.001) as shown in Figure 2 with standardised factor loading ranging from 0.54 to 0.97.

The convergent and discriminant validity were tested. Convergent validity is the extent to which indicators of a specific construct converge or share a high proportion of variance in common [55]. Convergent validity can be estimated through factor loadings and average variance extracted (AVE) [55,56]. AVE was used to measure the degree each construct differed from other construct in the model.

Discriminant validity is the fact that a factor is truly different from other construct. Hair et al. recommends that AVE should be greater than maximum shared variance (MSV); the square root for a factor should be bigger than inter construct correlation; and AVE should be greater than average shared variance (ASV) [56,57]. Going by the recommended thresholds as shown in Table 6, the four factors from this study do not have any convergent and discriminant validity and reliability issues.

#### 3.2.3. Structural Model Assessment and Hypothesis Testing

Table 7 presents the means, standard deviations, and correlation coefficient for the study variables. All the variables are significantly correlated (*p* < 0.01).

The structural model assessment of the proposed model for this study was accessed using PLS-SEM in the computer program SmartPLS, because the main objective of the model is to predict that safety commitment and communication (SCC), hazard management (HM), safe site practice (SSP), and job competence (JC) measures characteristics of HRO in construction as hypothesised. PLS fit measures are mostly variance based and focus on the discrepancy between the observed values of the dependent variables and the values predicted by the model in question [50]. The coefficient of determination (*R*^2^ value) is the most commonly used measure to assess structural models. R^2^ is a measure of the model’s predictive power and is calculated as the squared correlation between a specific endogenous construct’s actual and predicted values [50]. *R*^2^ value ranges from 0 to 1, with higher levels indicating higher levels of predictive accuracy. (*R*^2^ values of 0.75, 0.50, or 0.25 for endogenous latent variables are described as substantial, moderate, or weak) [58].

The path coefficient and *p* values are also used in structural model assessment. The path coefficient represents the hypothesized relationship between the constructs, with standardized values approximately between −1 and +1. Path coefficients close to +1 indicate strong positive relationships [50]. The T-statistics is used to establish the significant of direct effect and relationship, T is significant when the T-value is greater than 1.96 [58].

**Figure 3:**
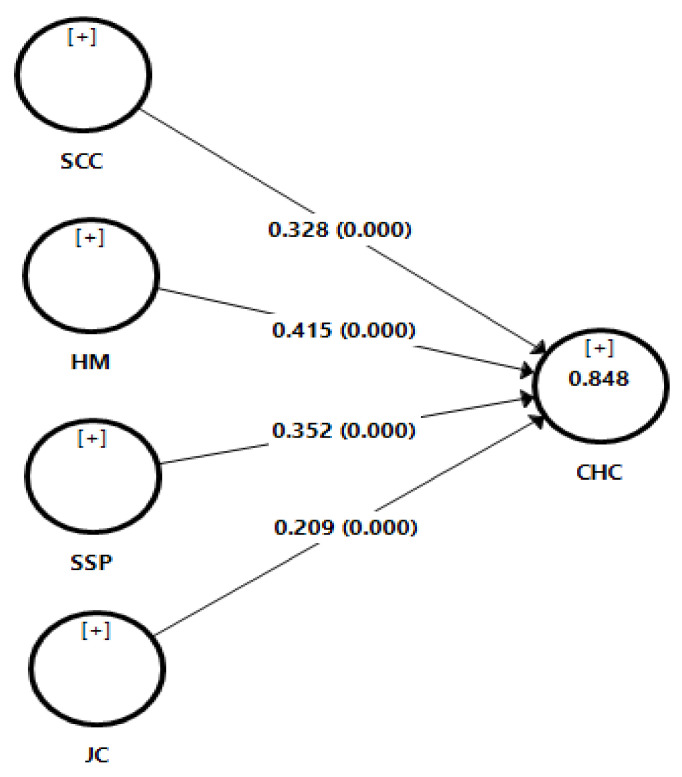
Structural model predicting Characteristics of HRO in Construction (CHC) (SCC—safety commitment and communication, HM—hazard management, SSP—safe site practice, JC—job competence).

The structural model assessment diagram is shown in Figure 3 and the assessment statistics in Table 8 and Table 9.

The structural model assessment revealed that the model had a high predictive power as *R*^2^ = 0.84, and there is a significant relationship and direct positive effect between SCC, HM, SSP, JC, and CH (*p* = 0.000). Additionally, *R*^2^ is above the recommended substantial threshold of 0.75 [58] and all T-statistics are larger than 1.96.

The hypothesis testing result is presented in the next paragraph.

*Hypothesis 1* proposed that perception of construction workers safety commitment and communication (SCC) predicts characteristics of HRO in construction (CHC), which is supported by the findings of this study. There was a significant positive direct effect of safety commitment and communication (SCC) on characteristics of HRO in construction (CHC). Therefore, hypothesis 1 was supported.

*Hypothesis 2* proposed that perception of construction workers hazard management (HM) predicts characteristics of HRO in construction (CHC), which is supported by the findings of this study. There was a significant positive direct effect of hazard management (HM) on characteristics of HRO in construction (CHC). Therefore, hypothesis 2 was supported.

*Hypothesis 3* proposed that perception of construction workers safe site practice (SSP) predicts characteristics of HRO in construction (CHC), which is supported by the findings in this study. There was a significant positive direct effect of safe site practice (SSP) on characteristics of HRO in construction (CHC). Therefore, hypothesis 3 was supported.

*Hypothesis 4* proposed that perception of construction workers job competence (JC) predicts characteristics of HRO in construction (CHC), which is supported in this study. There was a significant positive direct effect of job competence (JC) on characteristics of HRO in construction (CHC). Therefore, hypothesis 4 was supported.

The hypothesis has significantly proven that safety commitment and communication, hazard management, safe site practice, and job competence can measure and predict the characteristics of HRO in the construction industry.

## 4. Discussion

The aim of this study was to investigate how key safety management factors can be used to measure the characteristics of HRO in the construction industry in New South Wales Australia. Previous research by Harvey et al. [5] identified the barriers of transferring HRO utility to construction industry, they argued that according to normal accident theory, construction is not seen as a high risk industry despite the continuous growth in complex projects, but also suggested that today’s construction projects are more complex than what they use to be, indicating an opportunity to reconceptualise ‘high risk’ industries. In line with this, recent research have started exploring avenues to transfer HROs concepts to construction industry [17], and this study is one of such.

The findings of this study have empirically identified safety commitment and communication, hazard management, safe site practice, job competence as constructs that can effectively measure the characteristics of HRO. While previous studies conducted in the construction industry have been mostly qualitative [17]. This study addresses that gap by validating a measurement instrument and testing a model in the construction industry.

Safety commitment and communication accounts for an important aspect of safety management in every construction organisation, as it is very vital to provide a safe working environment. This usually involves the implementation of procedures and strategies to keep the working environment safe. Safety information is usually provided by management to workers via a two way communication of giving and receiving of information on work related hazards and risk controls. Safety commitment and communication was found to have a significant direct positive effect with CHC and can also measure and predict CHC. The statistical significance of SCC empirically identifies the presence of preoccupation with failure, reluctance to simplify, and sensitivity to operations.

Hazard management had a strong significant positive direct effect on CHC with a path coefficient of (0.415) from the structural model assessment, and high standardised estimates (β = 0.61, 0.65, 0.81, 0.83) on the measurement model. This finding indicates that construction organisations are committed to the management of workplace safety, and it can also be compared with the mindfulness study in Australia that identified construction workers as being more mindful of workplace health and safety based on their job role [13]. The findings can be attributed to the fact that construction activities are carefully planned and executed because of the risk involved, and the likelihood of workers being harmed from accidents as a result of unsafe act or unsafe working conditions. Therefore, to effectively manage safety, the industry makes use of risk assessment, hazard identification, supervision, and inspection to identify unsafe working conditions, work related hazards, and encourage workers to report safety concerns, incidents, and near misses. Hazard management direct positive significance identifies and measures preoccupation with failure, sensitivity to operation, commitment to resilience, and deference to expertise.

Safe site practice had the strongest significance and direct positive effect both for the hypothesis testing and instrument testing (standardized β = 0.54, 0.94, 0.97), and the highest T-statistics with a value of (13.07). Safe site practice involves more frontline managers as it has to with supervision and ensuring all safety procedures are followed. SSP identified and measured sensitivity to operation, commitment to resilience, and deference to expertise.

Job competence also had a significant positive direct effect on CHC though with the lowest path coefficient and T-statistics (0.048, 4.376) but had a relative high standardised estimate (standardized β = 0.73, 0.87, 0.93), and least mean score. This is mostly handled by management, as it involves ensuring workers have the right skills and experience to do their jobs. This measured reluctance to simplify, commitment to resilience and deference to expertise.

Research by Hoyland et al. [17] identified sensitivity to operation and commitment to resilience as the most prevalent HRO safety principle present in the construction industry in Norway, while deference to expertise was the least prevalent. The results from this study also identified deference to expertise as the least measured HRO characteristics under job competence, while sensitivity to operation, preoccupation with failure were the most measured under safety commitment and communication, hazard management, and safe site practice.

The results from the hypothesis testing have provided statistically significant evidence that characteristics of HRO can be measured in the construction industry through safety commitment and communication, hazard management, safe site practice, and job competence. The factor analysis also provided evidence that factors measure the characteristics of HRO, as this was further proven by the CFA, and all the variables were correlated. The measurement instrument had a good model fit with excellent goodness of fit (GFI), and the structural model had an above substantial R^2^ value (0.848). This study has empirically identified SCC, HM, SSP, and JC as constructs that measure and predict the characteristics of HRO (preoccupation with failure, reluctance to simplify, sensitivity to operation, commitment to resilience, and deference to expertise) present in the construction industry in Australia.

### 4.1. Implication for Construction Safety Management

Applying the aspects of HRO to other organisations has been a major challenge as some have argued that such can only be applied in extremely hazardous organisations, where their operations are tightly coupled [5]. Over the years, the construction industry has evolved and has become more complex with some similarities to HRO. However, despite the similarities, the industry struggles to find the balance between safety and production performances, making it difficult to avoid accidents, and achieve nearly error free operations. The findings from this paper have two practical implications for improving construction safety management.

The first implication is the identification and measurement of HRO characteristics present in the construction industry through the validation of a measuring instrument and testing a model that predicts characteristics of HRO. This evidence signifies the possibility of applying and transferring HRO attributes to the construction industry.

The second implication is that construction organisations can use the constructs (SCC, HM, SSP, and JC) that measure HRO characteristics to improve safety management as they have become more complex due to the increase in demand for commercial and residential buildings. This complexity is as a result of using advanced technology, machines, and subcontractors to meet demands and deadlines [59]. Construction organisations should ensure all relevant and safety critical information is effectively communicated to the people doing the job. Incident and near miss reporting should be encouraged as such information offers opportunity for learning and improvement; this can be achieved by establishing effective communication procedures and a strong learning culture. Construction sites are known to constantly change due to design alteration, weather conditions, or pressure to meet project deadlines, therefore organisations should not get complacent and allow safety to compete with production performance, rather they should be committed to putting safety first in their activities, learning from past events to improve safety performance, have a flexible decision making approach that values expertise and experience over rank, while effectively communicating safety information across the board.

### 4.2. Research Limitations

This study identified and measured characteristics of HRO present in the construction industry in New South Wales Australia, but there are some empirical limitations to be considered and followed up with future research. The study focused on a small number of construction organisations in a particular region in Australia, this was due to the fact that most construction organisations declined to allow their staff to take part in the study, so the results may not be generalizable. This research is a step in the right direction and a foundation for other research to build on. Additionally, the results can be used as a strategy to improve safety management of some high risk and general construction activities. Future research should focus of replicating studies like this with a broader population of construction organisations, so that the results can be compared and generalized.

## 5. Conclusions

The aim of this research was to provide empirical evidence on the measurement of the characteristics of HRO in the construction industry in New South Wales, Australia. From the analysis, safety commitment and communication, hazard management, safe site practice, and job competence were identified as constructs that measure the characteristics of HRO present in the system of work in the construction industry in Australia. The findings also present evidence that worker perception towards workplace safety increases as they move up the organisational hierarchy. This was evident in the domain scores as frontline managers had higher scores compared to frontline workers. The findings from this research will help facilitate knowledge transfer from HROs to contemporary high risk organisations such as construction, and also help improve construction safety management.

## Figures and Tables

**Table 1:** Participants by job role and staff type.

Job Role	ƒ	%
Frontline Managers		
Safety Officer	16	7.60
Site Supervisor	59	28.10
Foreman	14	6.70
Frontline Workers		
Metal worker	5	2.40
Welder	15	7.10
Scaffolder	7	3.30
Carpenter	32	15.20
Plant operator	4	1.90
Painter	2	1.00
Plumber	14	6.70
Plasterer	12	5.70
Demolition worker	4	1.90
Joiner	26	12.40
Total	210	100.00
Staff Type	ƒ	%
Middle Management	89	42.40
Frontline workers	121	57.60

**Table 2:** Participants by industry and organisation experience.

Years of Experience	In Organisation		In Construction	
	ƒ	%	ƒ	%
<5 years	107	51.00	84	40.00
5–10 years	80	38.10	68	32.40
11–15 years	14	6.70	35	16.70
16–20 years	3	1.40	8	3.80
>20 years	6	2.90	15	7.10
Total	210	100.00	210	100.00

**Table 3:** Participants by organisation and staff type.

Organisations/Type	Type of Project	Middle Management	Frontline	Total
Organisation 1 (Large)	High rise commercial buildings	18	28	46
Organisation 2 (Large)	Multiple blocks of residential buildings	16	20	36
Organisation 3 (Large)	Community shopping mall	17	23	40
Organisation 4 (Medium)	New Residential buildings	9	12	21
Organisation 5 (Medium)	Renovation of office buildings	10	13	23
Organisation 6 (Medium)	New Residential buildings	9	11	20
Organisation 7 (Medium)	New residential buildings	10	14	24
Total		89	121	210

**Table 4:** Summary of survey scores.

Variable Scores	Staff Group	Mean	Percent (%)
Safety Commitment and Communication (SCC)	Frontline managers	47.69	44.4
	Frontline workers	43.93	55.6
Hazard Management (HM)	Frontline managers	28.97	43.8
	Frontline workers	27.37	56.2
Safe Site Practice (SSP)	Frontline managers	30.08	43.7
	Frontline workers	28.52	56.3
Job Competence (JC)	Frontline managers	12.29	43.6
	Frontline workers	11.68	56.4
Characteristics of HRO in Construction Scores (SCC + HM + SSP + JC)	Frontline managers	119.05	44.0
	Frontline workers	111.51	56.0

**Table 5:** Rotated structure matrix for Principal Component Analysis (PCA) with Promax rotation for safety management constructs.

Scale	Components			
	1	2	3	4
SCC1		**0.715**		
SCC2		**0.725**		
SCC3		**0.705**		
SCC5		**0.640**		
SCC8		**0.413**		
SCC10		**0.493**		
SCC11		**0.543**		
HM1	**0.848**			
HM2	**0.883**			
HM3	**0.863**			
HM6	**0.514**			
HM7	**0.532**			
SSP1			**0.907**	
SSP2			**0.884**	
SSP3			**0.706**	
SSP4			**0.662**	
JC1				**0.447**
JC2				**0.960**
JC3				**0.946**
% Variance accounted for	27.638	10.328	9.201	8.873
Cumulative variance	27.638	37.967	47.168	56.040

Extraction method is principal component analysis, rotation method; Promax with Kaiser normalization. Factor loadings greater than 0.40 are in bold.

**Table 6:** Convergent and discriminant validity of factors.

Scale	CR	AVE	MSV	MaxR(H)	Safety_CC	Job_C	Safe_SP	Hazard_M
Safety_CC	0.784	0.549	0.157	0.800	0.741			
Job_C	0.885	0.722	0.100	0.917	0.317	0.850		
Safe_SP	0.873	0.707	0.126	0.959	0.276	0.269	0.841	
Hazard_M	0.820	0.537	0.157	0.846	0.396	0.266	0.355	0.733

**Table 7:** Descriptive statistics and correlation among variables.

Scale	M	SD	SCC	HM	SSP	JC	CHC
SCC	3.572	0.182	1	0.341 **	0.332 **	0.283 **	0.696 **
HM	4.006	0.242	0.341 **	1	0.335 **	0.211 **	0.786 **
SSP	3.585	0.174	0.332 **	0.335 **	1	0.252 **	0.684 **
JC	3.493	0.136	0.283 **	0.211 **	0.252 **	1	0.538 **
CHC	7.334	0.258	0.696 **	0.786 **	0.684 **	0.538 **	1

SCC: safety commitment and communication, HM: hazard management, SSP: safe site practice, JC: job competence, CHC: characteristics of HRO., M = mean, S = standard deviation. ** Correlation is significant at 0.01 level.

**Table 8:** Structural model assessment.

Hypothesis	Relationship					95% CI		
	Direct Effect	Path Coef.	St. Dev.	T-Statistics	*p*-Values	Lower	Upper	Decision
H1	SCC -> CHC	0.328	0.044	7.484	0.000	0.229	0.401	Supported
H2	HM -> CHC	0.415	0.034	12.325	0.000	0.346	0.479	Supported
H3	SSP -> CHC	0.352	0.027	13.078	0.000	0.300	0.406	Supported
H4	JC -> CHC	0.209	0.048	4.376	0.000	0.102	0.282	Supported

**Table 9:** *R*^2^ Statistics.

Factor	Path Coef	St. Dev.	T-Statistics	*p*-Values	Lower	Upper
CHC	0.848	0.059	14.435	0.000	0.720	0.953
